# Identification and validation of diagnostic cut-offs of the ELISpot assay for the diagnosis of invasive aspergillosis in high-risk patients

**DOI:** 10.1371/journal.pone.0306728

**Published:** 2024-07-09

**Authors:** Francesca Bettelli, Daniela Vallerini, Ivana Lagreca, Patrizia Barozzi, Giovanni Riva, Vincenzo Nasillo, Ambra Paolini, Roberto D’Amico, Fabio Forghieri, Monica Morselli, Valeria Pioli, Andrea Gilioli, Davide Giusti, Andrea Messerotti, Paola Bresciani, Angela Cuoghi, Elisabetta Colaci, Roberto Marasca, Livio Pagano, Anna Candoni, Johan Maertens, Pierluigi Viale, Cristina Mussini, Rossella Manfredini, Enrico Tagliafico, Mario Sarti, Tommaso Trenti, Russell Lewis, Patrizia Comoli, Albino Eccher, Mario Luppi, Leonardo Potenza

**Affiliations:** 1 Section of Hematology, Department of Medical and Surgical Sciences, University of Modena and Reggio Emilia, AOU Modena, Modena, Italy; 2 Diagnostic Hematology and Clinical Genomics, Department of Laboratory Medicine and Pathology, AUSL/AOU Modena, Modena, Italy; 3 Statistic Unit, Department of Medical and Surgical Sciences, University of Modena and Reggio Emilia, AOU Modena, Modena, Italy; 4 Hematology Division, Dipartimento di Diagnostica per Immagini, Radioterapia Oncologica ed Ematologia, Fondazione Policlinico Universitario A. Gemelli - IRCCS and Università Cattolica del Sacro Cuore, Rome, Italy; 5 Department of Hematology, University Hospital UZ Leuven, Leuven, Belgium; 6 Infectious Diseases Unit, Department of Medical and Surgical Sciences, University of Bologna, IRCCS-AOU Policlinico Santorsola, Bologna, Italy; 7 Clinic of Infectious Diseases, Dipartimento Chirurgico, Medico, Odontoiatrico e di Scienze Morfologiche con interesse Trapiantologico, Oncologico e di Medicina Rigenerativa, University of Modena and Reggio Emilia, AOU Modena, Modena, Italy; 8 Department of Biomedical, Metabolic and Neural Sciences, University of Modena and Reggio Emilia, Modena, Italy; 9 Department of Molecular Medicine, University of Padua, Padua, Italy; 10 Pediatric Hematology/Oncology Unit and Cell Factory, Foundation IRCCS Policlinico San Matteo, Pavia, Italy; 11 Section of Pathology, Department of Medical and Surgical Sciences, University of Modena and Reggio Emilia, AOU Modena, Modena, Italy; Leibniz-Institut fur Naturstoff-Forschung und Infektionsbiologie eV Hans-Knoll-Institut, GERMANY

## Abstract

**Objective:**

We investigated the performance of enzyme linked immunospot (ELISpot) assay for the diagnosis of invasive aspergillosis (IA) in high-risk patients with hematologic malignancies.

**Methods:**

We prospectively enrolled two cohorts of patients undergoing intensive myelosuppressive or immunosuppressive treatments at high risk for IA. ELISpot was performed to detect *Aspergillus*-specific T cells producing Interleukin-10.

**Results:**

In the discovery cohort, a derived cut-off of 40 spot forming cells (SFCs)/10^6^ PBMCs has shown to correctly classify IA cases with a sensitivity and specificity of 89.5% and 88.6%, respectively. This cut-off is lowered to 25 SFC when considering the subset of possible IA patients, with sensitivity and specificity of 76% and 93%, respectively. The application of the 40 SFCs cut-off to the validation cohort resulted in a positivity rate of 83.3% in proven/probable cases and a negativity rate of 92.5% in possible/non-IA cases. Adopting the 25 SCFs cut-off, the assay resulted positive in 83.3% of proven/probable cases while it resulted negative in 66.7% of possible/non-IA cases.

**Conclusions:**

ELISpot shows promises in the diagnosis of IA and the possibility to use two distinct cut-offs with similar diagnostic performances according to patients’ different pre-test probability of infection can widen its use in patients at risk.

## Introduction

The survival of patients with invasive aspergillosis (IA) has improved in the last few years, due to a wider antifungal armamentarium and a larger use of non-cultural based diagnostic methods (NCBDM) [1, 2]. Despite these major progresses, IA remains a major cause of morbidity and mortality for immunosuppressed patients [3]. An accurate diagnosis of active *Aspergillus* infection is eagerly required to initiate a timely and adequate targeted antifungal therapy. The conventional diagnostic methods, namely histopathologic evaluation and cultures are rarely feasible and often time consuming [4, 5]. The European Organization for Research and Treatment of Cancer and Mycoses Study Group (EORTC/MSGERC), recently updated the definitions of IA trying to overcome those drawbacks, and definitely incorporated polymerase chain reaction (PCR) as a diagnostic tool in addiction to galactomannan (GM) antigenemia [6, 7].

Several studies have clearly shown that adaptive immunity plays a crucial role in the host defense against filamentous fungi. In the murine model of invasive pulmonary aspergillosis, resistance to the infection was associated with the activation of CD4+ Th1 cells mainly producing Interferon-γ (IFN-γ), while the production of Interleukin-4 (IL-4) and Interleukin-10 (IL-10), by Th2 and Treg cells respectively, favor disease progression [8–10].

In PBMCs culture supernatants stimulated with *Aspergillus* antigens from hematologic patients with IA, a favorable response to antifungal therapy was found to correlate with a higher IFN-γ/IL-10 ratio while a progressive or stable disease correlated with lower IFN-γ/IL-10 ratio, supporting the hypothesis that the emergence of Th1 responses contributes to the host defense against *A*. *fumigatus*, while specific immune responses predominantly producing IL-10 may be non-protective to IA [11].

In healthy subjects, it has subsequently been demonstrated that T cells may proliferate and produce different amount of IL-10, IFN-γ, IL-4 and Interleukin-17A (IL-17A) in response to *Aspergillus* recombinant antigens and that the protective T cells may be expanded from their peripheral blood as possible source of adoptive therapy [12–14].

We have previously shown that *Aspergillus*-specific, *Mucorales*-specific and *Fusarium*-specific T cells may be detected in peripheral blood of patients with IA [15, 16], invasive mucormycosis [17, 18] and fusariosis [19], respectively, by means of ELISpot assay. Also in our in vivo data at the onset of infection and at progression the immune response to invasive fungal infection is polarized towards the production of IL-10, while the resolution of infection is associated with increasing IFN-γ production.

Then we have demonstrated that the detection of IL-10-producing *Mucorales-*specific T cells may be harnessed to diagnose invasive mucormycosis in hematological patients [17, 18].

Lately, others have reported that the cytofluorimetric occurrence of CD154+ mould specific T cells in peripheral blood, may be used as a supportive biomarker to diagnose invasive mould infections and to monitor therapeutic outcomes [20–24].

Moving from these premises, our current study proposes to investigate and validate the diagnostic performance of *Aspergillus*-specific T cells, detected by means of ELISpot assay, for the diagnosis of IA in high-risk patients with hematologic malignancies.

This study was designed as a development and validation multicenter study, enrolling hematologic patients undergoing intensive myelosuppressive or immunosuppressive treatments at high risk for IA. We prospectively recruited 2 patients’ cohorts (206 patients were enrolled in the discovery cohort and 100 patients in the validation cohort) and evaluated the potential diagnostic role of IFN-γ- and IL-10-secreting *Aspergillus*-specific T cells.

## Methods

### Patients

The study was a development and validation multicenter study, enrolling patients undergoing intensive myelosuppressive or immunosuppressive treatments at high risk for IA, between 01/07/2013 and 31/12/2019.

The study has been approved by the University of Modena and Reggio Emilia ethical committee (protocol number 63/11 CE AVEN, amendment n°2 of 03/06/2013) and written informed consent was obtained from each patient, according to the Declaration of Helsinki.

Patients were prospectively recruited because they showed clinical, radiological and/or microbiological factors suggestive of infectious processes. At the admission, they were screened at least with chest X-rays and, at the onset of fever, they underwent a diagnostic work-up to define the site and the etiology of the infectious/inflammatory process. The diagnostic work-up included culture of blood, urine, feces, molecular and serologic methods for the search of the most common viral pathogens, pulmonary and/or abdominal computed tomography (CT) scan, bronchoalveolar lavage (BAL) and biopsy of the affected tissue, when the latter procedure was allowed by the patients’ clinical conditions. BAL fluid was cultured for bacterial and fungal pathogens, mycobacteria, and *Legionella*, and was studied with immunofluorescent staining for *Pneumocystis jirovecii*, and molecular and cultural analysis for viral pathogens. The GM antigenemia (Bio-Rad Platelia^®^ *Aspergillus* EIA–Bio-Rad, Hercules, CA, United States), performed as described by the manufacturer, was detected twice a week in serum and, when performed, in BALf of acute leukemia and alloSCT patients, and, at discretion of the attending physician, in all the other patients. An index ≥0.5 for serum and ≥1.0 for BALf, were considered positive, respectively. The classification of fungal infection in the validation cohort was reassessed after the publication of new EORTC/MSGERC criteria^5^, and thus used different cut-off values particularly for concomitant positivity of GM detected in serum and BAL fluid and in single serum or plasma GM antigenemia. Patients were also surveyed for the development of sinonasal and cerebral infections. At the occurrence of symptoms suggestive of such infections, head CT and/or magnetic resonance imaging scans were also obtained. Autopsy of patients who died during the study period was obtained, when possible. Specimens of the organs involved by the infectious process were sent for either histological or cultural analysis. Hematoxylin-eosine, periodic acid-Schiff, Grocott or Gomori methenamine silver stains were used for the detection of hyphal invasion on tissue samples. The efforts to obtain the most certain diagnosis of the patients’ infectious disease were required to all clinicians, who enrolled patients. Antifungal prophylaxis was administered to high-risk patients.

### Sample collection and PBMCs isolation

After obtaining the informed consent, 30 ml whole peripheral blood samples in sodium citrate were collected weekly from the patients when at least one of the following criteria was present: 1) the onset of neutropenia; 2) the onset of fever; 3) the detection of radiological abnormal findings suggestive for infections; 4) the obtainment of a diagnosis of proven infection.

Peripheral blood mononuclear cells (PBMCs) were obtained by Lymphoprep^™^ (Alere Technologies AS) gradient centrifugation. PBMCs were suspended in 1 ml of R10 medium (RPMI 1640 + 10% FBS, 1% Ampicillin, 0.5% Gentamicin, 1% Sodium Pyruvate), counted using an automated cell counter (AcT8, Beckman Coulter) and cryopreserved (S1 and S2 Files).

### Enzyme linked immunospot (ELISpot) assay

The detection of *Aspergillus*-specific T cells producing IFN-γ and IL-10 was performed by enzyme linked immunospot (ELISpot) assay, as previously reported [15–19] and detailed below. ELISpot has been performed by 3 of the authors (D.V., P.B. and I.L.), having high experience with this assay, and who were unaware of the clinical condition of the patients.

A total of 100.000 viable cells/well were cultured on 96-well plates pre-coated with anti-cytokine antibodies (Mabtech), namely anti-IFN-γ and anti-IL-10, for 18 or 40 hours respectively. As antigen stimulation we used heat-killed germinated conidia after four cycles of sonication at 280 W with a frequency of 24 KhZ, from fungi of the *Aspergillus fumigatus* species, at a concentration of 200.000 conidia/ml. Unstimulated PBMCs were used as negative controls, whereas anti-CD3 antibody (Mabtech) was added to positive control wells. After incubation, ALP-conjugated antibodies anti-IFN-γ or anti-IL10 were added, and plates were then processed according to manufacturer’s instructions.

The number of spot forming cells (SFCs) per well was quantified using an automated ELISpot counter (AID-GmbH, Strassberg, Germany). All test conditions were carried out in triplicate and results for individual samples were calculated as a median value of SFCs obtained in antigen-stimulated wells compared to control wells and expressed as number of SFCs on 10^6^ PBMCs.

A sample was considered positive if all the followings were fulfilled: 1) the presence of at least 5 SFCs; 2) the presence of more than 5 SFCs in the sample compared with the negative control; 3) the presence of a stimulation index of ≥2 (defined as ratio of SFCs in the sample versus the negative control). Patients with two or more samples were considered positive if ≥50% of the analyzed samples resulted positive. Moreover, samples were considered not informative/undetermined when they were not responsive to anti-CD3 stimulation in positive control wells.

The accuracy of our laboratory in the execution of ELISpot assay is periodically evaluated by participating in the IMMUDEX proficiency panel, an external validation program of assay performance on cryopreserved samples, always reaching scores in the “average range”.

### Statistical analysis

Student’s T test was used to compare the mean number of *Aspergillus*-specific T cells producing IFN-γ and IL-10 in patients with proven IA and controls.

Percentages of the tests with ≥100 SFCs/10^6^ were determined and compared between the two group of patients, by using Fisher’s exact test.

Sensitivity, specificity, positive predictive values (PPV), negative predictive values (NPV) of the ELISpot assay for the diagnosis of IA have been calculated. Sensitivity was calculated from the results of proven IA cases, specificity from those of the control group. The PPV, NPV and efficacy were estimated from the combination of both groups. 95% confidence intervals (CI) have been calculated for all the above-mentioned estimates. A receiver-operating characteristic (ROC) analysis was performed to determine the best cut-off level that yielded the maximum sensitivity plus specificity. Considering that measurements were performed repeatedly, the highest values were used for the ROC analysis.

Moreover, to reduce the bias related to real-life experience of most of clinical hematologists, who manage patients classifiable as possible IA in most cases, in our discovery cohort we evaluated the performance of the ELISpot assay in patients satisfying the criteria of possible IA at some point of their disease trajectory. For the final analysis the proven and probable cases were considered as positive cases while the possible IA and proven other than IA cases were considered as negative cases. We performed the ROC analysis also in this subset of patients.

In the validation cohort, the diagnostic accuracy of the identified cut-offs was performed by considering the proven and probable IA cases as positive cases while the possible IA and proven other than IA cases as negative cases.

Fischer’s exact test was used to compare the frequency of undetermined results between classes of patients with different WBC counts. A 2-sided *P* value of less than .05 was considered as statistically significant. The results were obtained using the Stata Software (10.0, College Station, Texas, USA).

## Results

### Patients’ cohorts

Two hundred and six patients were enrolled in the discovery cohort, namely 20 proven IA according to the revised EORTC-MSG criteria and 186 controls. Sixteen (80%) out of 20 proven IA patients were hematological patients and of these, 13 (65%) were affected by acute leukemias. The median age was 49 years (range 17–78). One hundred fifty-nine (85.5%) out of 186 controls were hematological patients, mainly receiving chemotherapy for acute leukemias (69.9%). The median age was 57 years (range 12–81). One hundred sixteen patients (62.4%) were receiving antifungal prophylaxis. The clinical characteristics of the patients are reported in Table 1 and S1 Table.

**Table 1:** Clinical characteristics of 186 control patients from the discovery cohort.

**Median age (y)**	57 (12–81)
**Acute myeloid leukemia**	103 (55.4)
**Acute lymphoblastic leukemia**	27 (14.5)
**Multiple myeloma**	10 (5.4)
**NHL**	19 (10.2)
**SOT**	2 (1)
**Other**	25 (13.4)
**Antifungal prophylaxis N° (%)**	116 (62.4)
**Allogeneic/autologous HSCT (%)**	4/8 (6.5)
**Site of Infection**	N° (%)
Lung	149 (80)
Liver	2 (1)
Other	35 (19)
**Etiology of the infection in 133/186 control patients**	N° (%)
Fungi (other than aspergillus infections)	14 (10.5)
Bacteria	76 (57.1)
Viruses	17 (12.8)
Other	26 (19.5)

The prospective validation cohort included 100 patients, namely 71 (71%) affected by acute myeloid leukemia (AML) and 17 (17%) affected by acute lymphoblastic leukemia (ALL). The median age was 52 (range 19–80). The clinical characteristics of the validation cohort are reported in Table 2. Fifteen (15%) out of 100 patients of the validation cohort showed radiological signs consistent with possible IA at the time of the first HRCT, namely dense, well-circumscribed lesions with or without a halo sign, air crescent sign, cavity, wedge-shaped and segmental or lobar consolidation. Of these, 1 had a final diagnosis of proven IA, 6 probable IA and 3 possible IA, after a complete diagnostic work-up, according to the definition of Invasive fungal disease from 2008 EORTC-MSG criteria [6]. The same patients were reassessed according to the revised thresholds for GM proposed by 2019 EORTC-MSG criteria [7], obtaining very similar results: 1 proven IA, 5 probable IA and 4 possible IA. In 5 out of these 15 patients the diagnosis of fungal infection was ruled out by microbiological findings. The remaining 85 patients showed clinical, microbiological and radiological features unlikely to be related to IA and were considered as controls.

**Table 2:** Characteristics of the 100 patients from the prospective validation cohort.

**Age (years), median (range)**	52 (19–80)
**Acute Myeloid Leukemia**	71 (71%)
**Acute Lymphoblastic Leukemia**	17 (17%)
**Lymphoma**	5 (5%)
**Severe aplastic anemia**	2 (2%)
**Other**	5 (5%)
**Neutropenia n° (%)**	91 (91%)
**Anti mould Prophylaxis n° (%)**	70 (70%)
**Site of Infection identified in 66/100 patients**	**N° (%)**
Lung	49 (74.2%)
GI	2 (3%)
Skin	3 (4.6%)
Bloodstream	8 (12.1)
Other	4 (6.1%)
**Etiology of the Infection/Inflammation in 53/100 patients**	**N° (%)**
Fungi	15 (10 aspergillosis*, 3 fusariosis and 2 candidiasis) (28%)
Bacteria	27 (51%)
Viral	11 (21%)
2019 EORTC/MSG Classification of IA (%) *	
Proven IA	1
Probable IA	5
Possible IA	4

All the patients were tested by the ELISpot assay. In the discovery cohort, a total of 62 (average 3 per patients; range from 1 to 8) and 450 (average 2 per patients; range from 1 to 8) PB samples were collected and analyzed from proven IA and control patients, respectively. In the validation cohort, the total number of analyzed PB samples was 277 (average 3 per patients; range from 1 to 5). Each sample was tested for the detection of both IFN-γ- and IL-10-producing T cells, resulting in a total of 1578 essays.

### *Aspergillus*-specific T cells for the diagnosis of IA

#### Discovery cohort

IFN-γ-ELISpot assay resulted informative in all tested samples, while IL-10-ELISpot assay was not informative in 4 (6.5%) out of 62 samples. When patients were considered, only one (0.5%) out of 20 was not informative for IL-10.

Overall, 19 (95%) out of 20 proven IA patients showed *Aspergillus*-specific T cell responses. In details, *Aspergillus*-specific T cells producing IL-10 were detected in 18 (94.7%) of 19 informative patients, and *Aspergillus*-specific T cells producing IFN-γ were found in 6 (30%) out of 20 patients. In the control group, 25 (5.5%) and 78 (17.3%) samples out of 450 resulted not informative in IFN-γ- and IL-10-ELISpot assay, respectively. The frequency of undetermined samples was not associated with patients’ WBC counts; indeed, no statistically significant associations were found when the rates of undetermined samples were compared in patients with different classes of WBC counts (0–500, 501–1000, 1001–2000, 2001–5000, 5000–10000, >10000 cells/cmm), being p value 0.493.

Overall, 11 (5.9%) out of 186 control patients were not informative for IL-10, while all patients were informative for IFN-γ. ELISpot assay showed the presence of *Aspergillus*-specific T cells producing IFN-γ in 14 (7.5%) out of 186 patients, while IL-10-producing *Aspergillus*-specific T cells were detected in 20 (11.4%) out of 175 cases.

When we compared control patients with patients with proven IA, it resulted that the magnitude of specific immune response, evaluated either as the number of samples with more than 100 SFCs or as mean SFCs, was significantly greater in proven patients. In particular, the number of IL-10 tests revealing >100 SFCs/10^6^ PBMCs was 26 (41.9%) out of 62 in the group of proven IA and 22 (4.9%) out of 450 in controls (p < .05). Regarding IFN-γ tests, only 4 (6.5%) out of 62 and none out of 450 revealed more than 100 SFCs/10^6^ PBMCs, in proven IA and control patients respectively.

**Fig 1:**
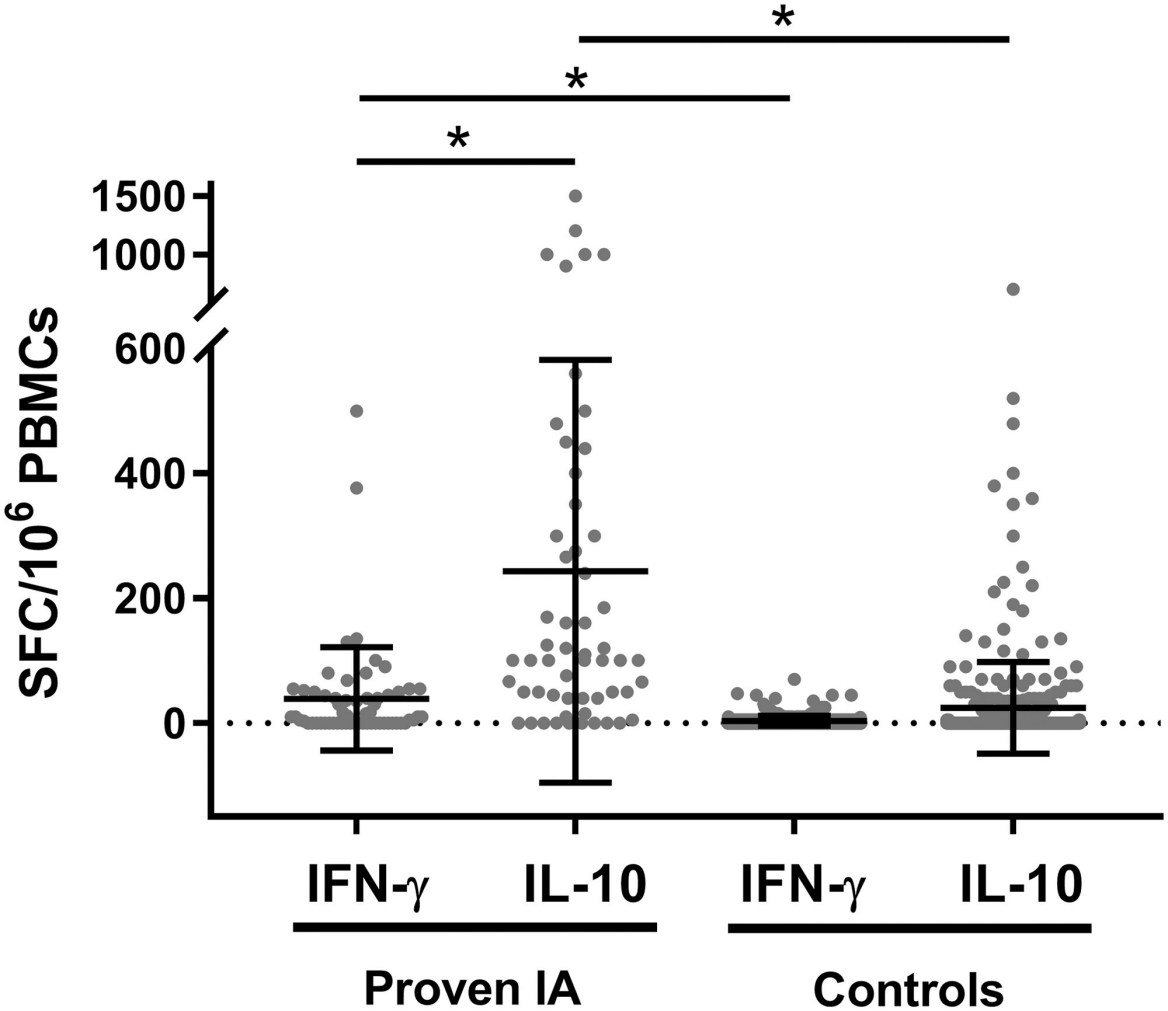
*A*. *Fumigatus*-specific T cell detection by ELISpot assay in patients with proven IA and controls, discovery cohort. Box plot showing immune responses producing IFN-γ and IL-10 in patients with proven IA (n = 20) and controls (n = 186). Individual data points for each patient are shown. Results are expressed as the number of SFC per 10^6^ PBMCs. Mean with SD is given. * = P<0.05.

Moreover, mean number of *Aspergillus*-specific T cells producing IFN-γ and IL-10 was significantly higher in proven patients compared to control ones (37.7 vs 3.6, p < .05; 234.7 vs 26.8, p < .05) (Fig 1).

Interestingly, the 20 patients with proven IA showed significantly higher mean number of *Aspergillus*-specific T cells producing IL-10 than those producing IFN-γ (234.7 vs 37.7, p < .05) (Fig 1). In addition, considering proven IA, the number of tests with more than 100 SFCs/10^6^ PBMCs was 26 (41.9%) and 4 (6.5%) out of 62 for the detection of IL-10 and IFN-γ, respectively (p < .05).

These data are in line with our previous findings, showing that at the onset of infection and during progression the kinetics of immune response to IA is polarized towards the production of non-protective cytokines (namely IL-10), while the resolution of infection is associated with increasing IFN-γ production [15].

**Fig 2:**
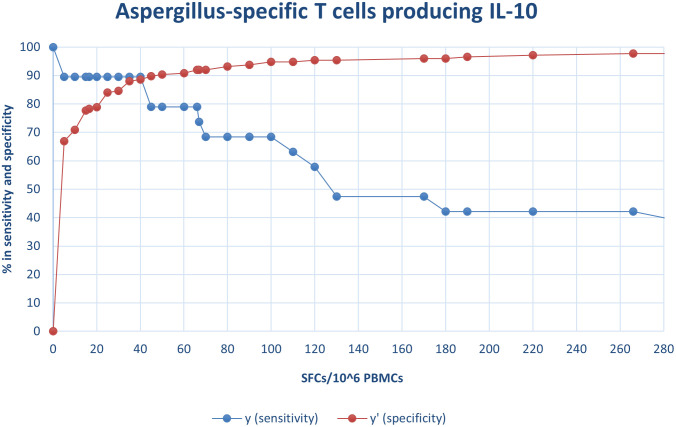
Receiver-operating characteristic (ROC) analyses to derive cut-offs. ROC analysis was performed between the cases with proven IA versus control group to determine the cut-off level that yielded the maximum sensitivity plus specificity, according to the presence of *Aspergillus*-specific T cells producing IL-10. aROC = 0.8953 (95% confidence interval: 0.80–0.99); optimal criterion ≥ 40; sensitivity: 89.5%; specificity: 88.6%. aROC = area under the ROC curve.

**Fig 3:**
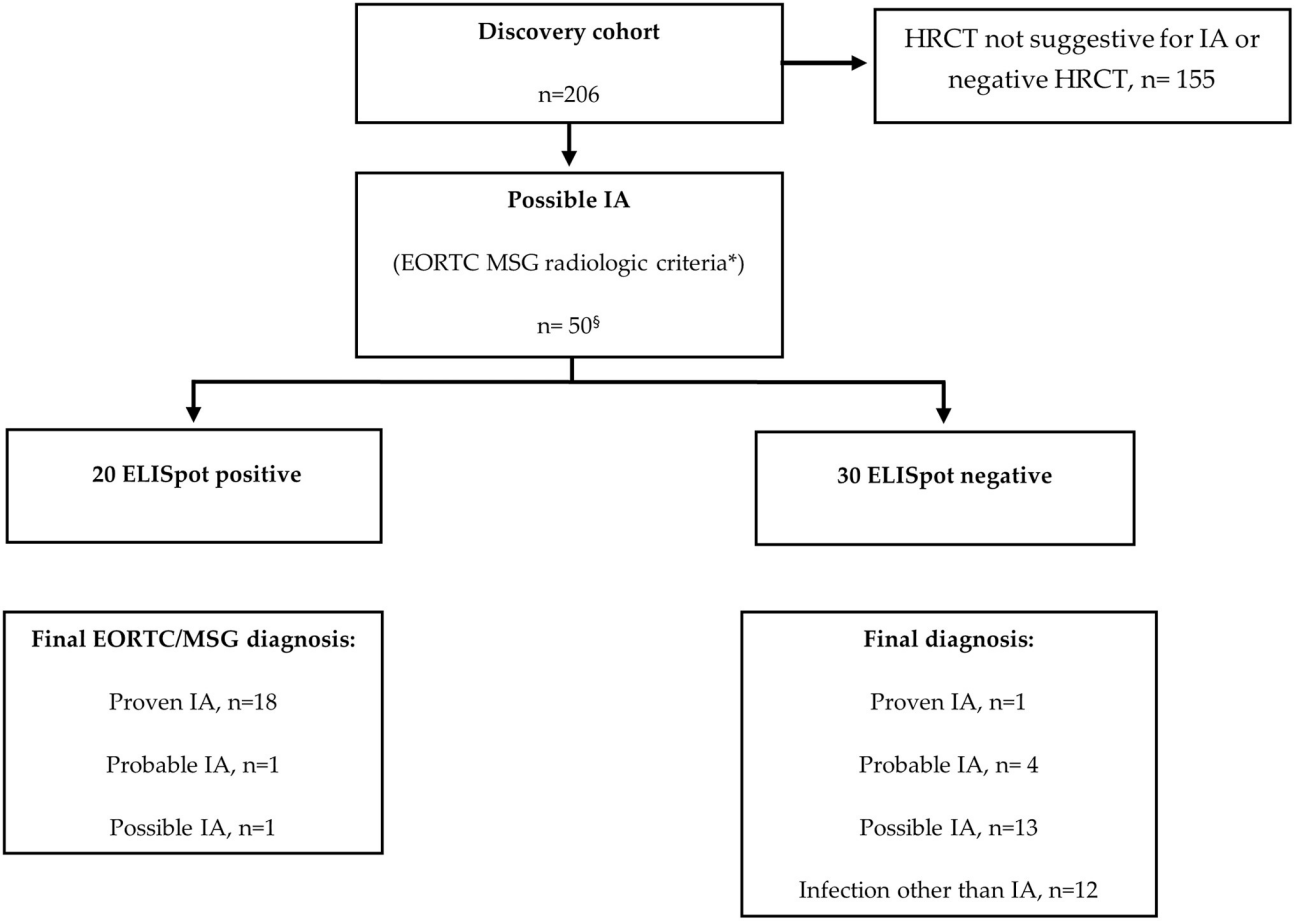
Study design flow chart, discovery cohort. Abbreviations: HRCT high resolution computed tomography; EORTC European Organization for Research and Treatment of Cancer; MSG Mycoses Study Group; SFCs Spot Forming Cells. * Dense, well-circumscribed lesions with or without a halo sign, air crescent sign, cavity, wedge-shaped and segmental or lobar consolidation. ^§^ One out of 51 samples excluded from the analysis because not informative.

By considering these premises, we performed a ROC analysis to investigate the potential of IL-10-ELISpot in the diagnosis of IA. The ROC analysis showed that the cut-off providing the best combination of sensitivity and specificity was obtained when at least 40 SFC/10^6^ PBMCs of *Aspergillus*-specific T cells producing IL-10 were detected. The former resulted 89.5% (95% CI, 66.9%-98.7%) and the latter 88.6% (95% CI, 82.9% -92.9%), respectively. The PPV of the test was 45.9% (95% CI, 29.5%-63.1%), the NPV was 98.7% (95% CI, 95.5%-99.8%) (Fig 2). Fifty-one (24.3%) out of 206 patients of the discovery cohort showed radiological signs consistent with possible IA at the time of the first HRCT according to the EORTC/MSG criteria. Of these, 19 had a final diagnosis of proven, 5 probable, 14 possible IA and the remaining 12 had evidence of infections other than IA (Fig 3). One patient out of 51 was not informative and thus it was excluded from the final analysis. In this subset of patients, ELISpot was positive in 18 (95%) out of 19 patients with a final diagnosis of proven cases and negative in all the 12 (100%) cases with a final diagnosis of bacterial or viral pneumonia. ELISpot resulted positive in one (20%) of 5 probable cases and one (7.1%) of the 14 possible cases with no alternative diagnosis. Of note, this latter patient had a clinical and radiological response to liposomal Amphotericin B (L-AmB) (Fig 3).

In this subset of possible IA patients, ROC curve showed that the cut-off with the best sensitivity and specificity was achieved with at least 25 SFC/10^6^ PBMCs of *Aspergillus*-specific T cells producing IL-10, being the former 76% (95% CI:59%-91%) and the latter 93% (95% CI: 76%-98%), respectively. The NPV was 80% (95% CI: 61.4%-92.3%) and the PPV was 91% (95% CI: 69.6%-98.8%).

#### Validation cohort

In the 100 patients enrolled in the Validation cohort, The ELISpot assay resulted positive for the presence of *Aspergillus*-specific T cells producing IL-10 in 20 (20%). When we applied the 40 SFCs cut-off, the patient positivity rate among the proven/probable patients was 83.3% (5 out of 6). Among negative patients (possible IA + controls), the negativity rate was 92.5% (87 out of 94). S2 Table shows the correlation between the ELISpot results and the clinical, radiological and NCBDM for all the 100 patients of the validation cohort.

Then in the group of 15 (15%) out of 100 patients showing radiological signs consistent with possible IA at the time of the first HRCT, we adopted the 25 SFCs cut-off. The assay confirmed positive results in 5 out of 6 (83.3%) proven/probable patients, namely the only proven case and 4 out of 5 probable cases. The assay resulted negative in 6 out of 9 (66.7%) negative patients, namely in 1 out of 4 possible cases and in 5 out of 5 patients for whom the diagnosis of fungal infection was eventually ruled out by microbiological findings (Table 3).

**Table 3:** Diagnostic concordance between 2019 EORTC/MSG and ELISpot.

2019 EORTC/MSG–based Diagnosis	ELISpot results	
	ELISpot positive (≥25 SFCs)	ELISpot negative (<25 SFCs)
Proven IA (n = 1)	Invasive Aspergillosis, n = 1	Not applicable
Probable (n = 5)	Probable IA, n = 4	Probable IA, n = 1
Possible (n = 4)	Possible IA, n = 3	Possible IA n = 1

**Fig 4:**
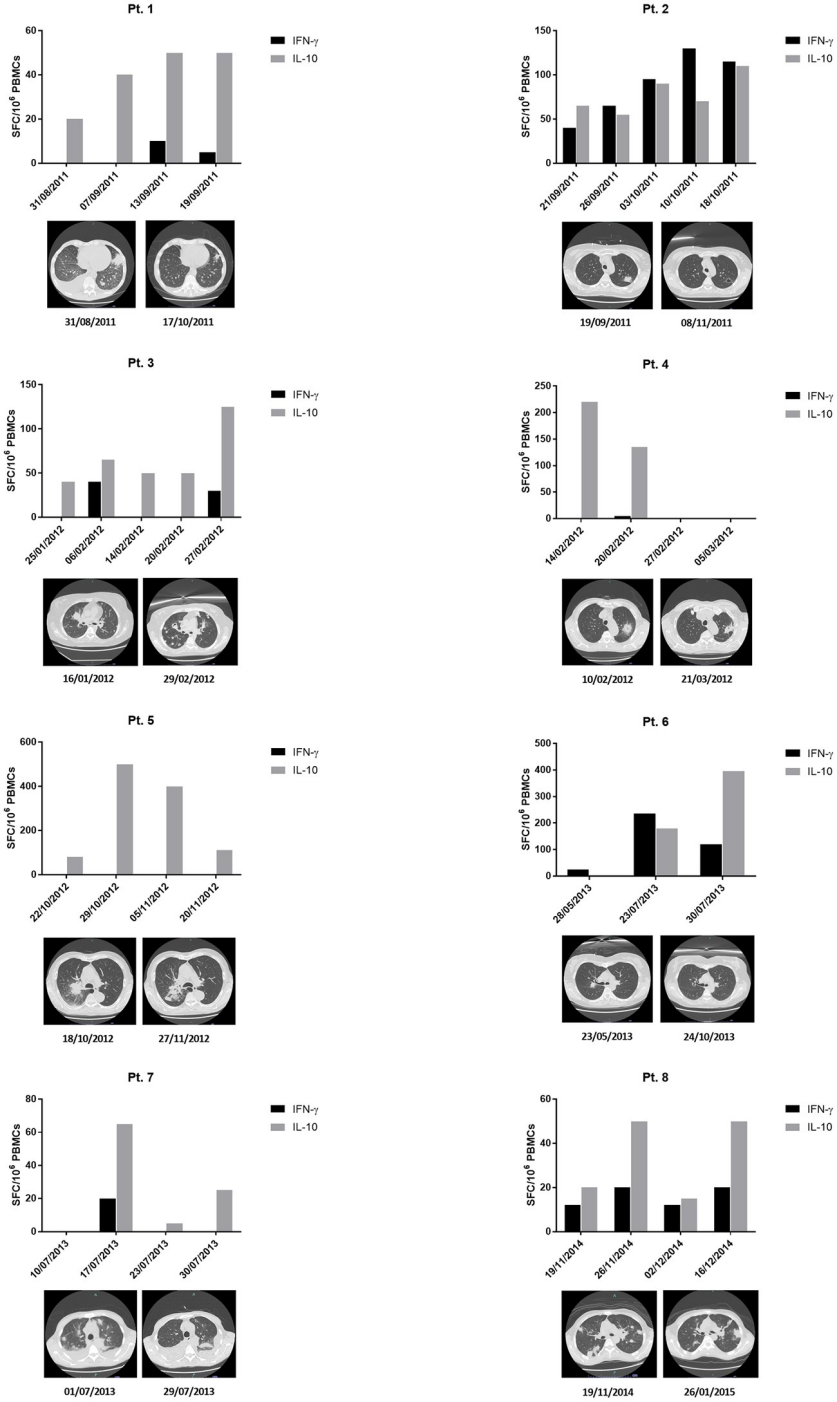
*A*. *fumigatus*-specific T-cell responses in patients showing ELISpot positivity. Column graphs represent the values of T cells producing IFN-γ and IL-10 at different time-points. Chest HRCT (High-Resolution Computed Tomography) images show the radiologic evolution during the course of the infection.

In those 8 patients showing ELISpot positivity, in Fig 4, we have detailed the individual kinetics of specific T-cell responses and its correlation with changes in radiologic signs during the course of the infection. Of note, 2 out of the 3 patients with possible IA resulting positive at the ELISpot assay showed cavitation of the pulmonary lesion in the follow up radiological studies and the remaining one resolved the pulmonary infiltrate with L-AmB.

Furthermore, IFN-γ- and IL-10-secreting *A*. *Fumigatus*-specific T cells were quantified by ELISpot assay as described in the Methods section, in five hematologic patients at high risk for invasive aspergillosis, comparing paired fresh and cryopreserved samples. Cell viability was evaluated both after thawing and after the resting step, with values of 92–94% and 80–88% on average, respectively. Differences were not significant according to Wilcoxon matched-pairs test, being p value 0.6250 and 0.4375 for IFN-γ and IL-10 ELISpot, respectively (S1 Fig).

## Discussion

As previously reported, IA patients, as well IM patients, may develop mould-specific immune responses predominantly polarized to the production of IL-10 at the onset of the infection, while the increase of protective T cells occurs later and is associated with a favorable outcome [15–18]. Thus IL-10 could be considered a signature of mould infection and could be exploited as a novel supportive biomarker in the diagnosis of invasive mycosis. Given its prominent effect on the resolution of inflammation, IL-10 production may be a consequence, rather than a cause, of the infection. However, as IL-10 production may limit the efficacy of protective immune responses, the consequence could be not only reduced damage to the host but also fungal persistence and eventually immunosuppression [10].

Based on these premises, the current study shows the potential of detecting *Aspergillus-*specific T cells producing IL-10 to diagnose IA in a large series of hematologic patients at high-risk for invasive fungal infections.

A derived cut-off of 40 SFCs has shown to correctly classify IA cases with a sensitivity and specificity of 89.5% and 88.6%, and NPV and PPV of 98.7% and 45.9%, respectively. The good performance of the assay should be considered because obtained on a valuable number of proven IA patients and histologically-culturally confirmed controls, within a patient population with a prevalence of IA of 10%, which could be well applicable to the general hematologic population [25, 26]. The application of this cut-off to the validation cohort resulted in a positivity rate of 83.3% in proven/probable cases and a negativity rate of 92.5% in possible/control cases.

Although the large number of proven cases is a strength of this study, it may also introduce a bias as, nowadays, most of the hematologists deal very often with possible IA cases. The analysis of patients classifiable as possible IA, at any time, during their infectious disease course, demonstrated that the high diagnostic accuracy of the assay may be maintained with a cut-off of 25 SCFs/10^6^ PBMCs. Such a threshold showed a sensitivity and specificity of 76% and 93%, and a NPV and PPV of 80% and 91%, respectively.

The application of the 25 SCFs/10^6^ PBMCs cut-off to the validation cohort, would have diagnosed the proven IA case and 4 out of 5 probable IA patients and would have given negative results in 5 (100%) out of 5 patients with proven diagnosis other than IA. Moreover, in this cohort, the assay would have upgraded 3 (33%) out of 9 possible cases (4 possible + 5 other than IA) reflecting the pretest probability of possible IA cases, which has been reported to be between 30% and 50% in the literature [27]. Of note, all three patients showed a clinical course consistent with IA, which was resolved with antifungal treatment. We believe these results are of relevance because obtained in a situation that further mirrors the real-life clinical experience.

The adoption of two different cut-offs may be justified by the fact that patients at high risk of IA have different probability of developing the disease at different time points during their at-risk period.

Moreover, it is in accordance with the Bayes’ Theorem which recognizes context as one of the most important factors in clinical decision making, in particular in infectious diseases. According to this theorem, a diagnostic test can best be interpreted when the pretest probability (prevalence) is known. For these reasons we would suggest that, to start antifungal treatment, clinicians treating patients at risk for IA, should consider the 40 SFCs/10^6^ PBMCs threshold when they deal with patients without pulmonary lesions or with pulmonary lesions who may undergo invasive procedure to achieve a final histological and cultural diagnosis. On the other hand, clinicians should consider the 25 SFCs/10^6^ PBMCs threshold when they deal with patients with pulmonary lesions who are very unlikely to receive a histological/cultural diagnosis of IA.

In summary, the ELISpot assay shows promises in the diagnosis of IA as it is sensitive, robust and easy to perform, even if trained personnel and the optimization of harmonized protocols are strongly recommended for using this assay in the diagnostic routine. As previously reported [15, 17–19], we used in-house heat-killed and sonicated conidia as antigenic stimulation, to provide a broader range of stimulation compared to the use of individual antigens. Although a concern may be a reduced standardization, it does not represent an inherent limitation of the ELISpot assay per se. However, commercial availability of quality-controlled mould specimens would be essential in order to improve the standardization of ELISpot assay across different laboratories.

Currently, alternative investigational assays based on whole blood protocols for the cytofluorimetric detection of mould-reactive T cells [22] or for ELISA-based detection of mould-induced T-helper cell signature cytokines [28] are under development, with the aim to reduce the impact of pre-analytical sample handling and hands-on time.

Actually, appropriate blood sample processing is essential to guarantee reliable functional T-cell assay performances. In particular, the time lag between blood collection and sample processing appears to be a critical parameter greatly affecting cell recovery and function [29, 30]. Of note, our study shows that immediate freezing of isolated PBMCs at the patient’s local center with subsequent storage and shipment to specialized laboratory is feasible and may contribute to overcome such challenge.

Our multicenter study was conducted exclusively on cryopreserved samples. Even if the use of cryopreserved PBMCs implies some variability, compared to fresh blood samples [30], cryopreservation should not be excluded. What matters for reliable results is cell quality, and relevant to this, samples should have a high proportion of viable cells and a low proportion of apoptotic or dead cells. Standard freezing and thawing procedures usually allow to preserve cell viability. Moreover, a resting step after thawing allows for removal of cell debris, improving assay performance. Actually, in our experience, cryopreservation doesn’t affect ELISpot performance and facilitates short-term storage and sample shipment to specialized laboratories.

However, further standardization of protocols for blood collection, sample storage, PBMCs isolation and shipment is required.

The possibility to use two distinct cut-offs with similar diagnostic performance suggests that ELISpot could be used to support the diagnosis of IA in all patients at risk, according to their different pre-test probability of the infection. Moreover, the high negative and positive predictive value also in a group of patients receiving concurrent azole prophylaxis, suggest that ELISpot assay may be useful as a complementary diagnostic tool also in such circumstances. By exploiting host factors, the ELISpot assay used in combination with other non-culture diagnostic methods could enable a more consistent diagnosis of IA in a diagnostic-driven work-up and could be used to improve the administration of proper antifungal therapy [30, 31].

However, further prospective studies are needed to define a multidimensional data analysis taking into account all the clinical variables affecting the performance of the assay (e.g., underlying disease, active chemotherapy, corticosteroids, antifungal prophylaxis that might affect antigen burden, co-infections etc.) and to identify the optimal sampling strategies in order to improve the feasibility of ELISpot in routine clinical setting (single time analysis rather than weekly monitoring). Moreover, the identification of the antigens eliciting the strongest protective responses in patients with IA, may spur further studies to define the complete repertoire of *Aspergillus* specific immune responses during the course of the infection and to design therapeutic strategies of either vaccine or autologous cytotoxic cell infusions.

## Supporting information

S1 Table Clinical characteristics of the 20 patients with proven IA. Abbreviations: AML = Acute Myeloid Leukemia; LLA = Acute Lymphoblastic Leukemia; CLL = Chronic Lymphoblastic Leukemia; NHL = non-Hodgkin’s lymphoma; SOT = Solid Organ Transplant; alloSCT = allogeneic hematopoietic stem cell transplant; neg = negative; pos = positive; n/a = not available; IA = invasive aspergillosis.
(DOCX)

S2 Table Clinical, radiological and microbiological characteristics of the validation cohort and sample collection. Abbrevations: Pos. = positive; uninf. = uninformative; HRCT = High-Resolution Computed-scan Tomography; AML = acute myeloid leukemia; MDS = myelodisplastic syndrome; HL = Hodgkin Lymphoma; NHL = non-Hodgkin lymphoma; ALL = Acute lymphoblastic leukemia; MM = multiple myeloma; ASCT = autologous stem cell tranplantation; alloSCT = allogeneic hematopoietic stem cell transplant; S. aureus = Staphylococcus aureus; NA = not applicable.
(DOCX)

S1 Fig *A*. *Fumigatus*-specific T cell detection by ELISpot assay in fresh and cryopreserved samples. Results are expressed as the number of SFC per 10^6^ PBMCs. Mean with SD is given.
(TIF)

S1 File PBMCs cryopreservation. (DOCX)

S2 File PBMCs thawing. (DOCX)
