# Supplementary material for: Identification and validation of diagnostic cut-offs of the ELISpot assay for the diagnosis of invasive aspergillosis in high-risk patients
Source: PLoS One. 2024 Jul 9;19(7):e0306728. doi: 10.1371/journal.pone.0306728 (PMC11233002; doi:10.1371/journal.pone.0306728)
Supplement: S1 Table — Abbreviations: AML = Acute Myeloid Leukemia; LLA = Acute Lymphoblastic Leukemia; CLL = Chronic Lymphoblastic Leukemia; NHL = non-Hodgkin’s lymphoma; SOT = Solid Organ Transplant; alloSCT = allogeneic hematopoietic stem cell transplant; neg = negative; pos = positive; n/a = not available; IA = invasive aspergillosis. (DOCX) [file pone.0306728.s001.docx]

| Proven IA | Sex/Age | Underlying Disease | Neutropenia | Infection  Site | GM Serum | GM BAL | Cultural  BAL | Cytology BAL | Histology | Cultural  Biopsy | Autopsy |
| --- | --- | --- | --- | --- | --- | --- | --- | --- | --- | --- | --- |
| PT1 | F/59 | AML | Yes | Lung | neg | neg | neg | neg | IA | *A. fumigatus* | n/a |
| PT2 | F/67 | AML | Yes | Lung | neg | pos | pos | pos | IA | *A. fumigatus* | n/a |
| PT3 | M/23 | ALL | Yes | Lung | n/a | pos | neg | neg | IA | *A. fumigatus* | n/a |
| PT4 | M/63 | CLL | No | Lung | n/a | pos | neg | neg | IA | *Aspergillus spp* | n/a |
| PT5 | M/67 | NHL | No | Lung | n/a | neg | neg | neg | IA | *A. fumigatus* | n/a |
| PT6 | M/15 | AML | No | Lung, Liver | n/a | pos | neg | neg | IA | *A.fumigatus* | n/a |
| PT7 | M/57 | AML | Yes | Lung | pos | pos | pos*/ A. fumigatus* | pos | IA | n/a | *Aspergillus spp* |
| PT8 | F/57 | SOT | No | Lung | neg | neg | pos/ *A.fumigatus* | pos | n/a | n/a | *Aspergillus spp* |
| PT9 | M/48 | ALL | Yes | Lung, Spleen | pos | pos | neg | neg | IA | *A.fumigatus* | n/a |
| PT10 | M/66 | AML | Yes | Lung | neg | pos | neg | neg | IA | n/a | *Aspergillus spp* |
| PT11 | F/54 | SOT | No | Lung | pos | pos | pos/ *A. fumigatus* | pos | n/a | n/a | *Aspergillus spp* |
| PT12 | M/65 | SOT | No | Lung | n/a | pos | pos/ *A.niger* | neg | IA | n/a | *Aspergillus spp* |
| PT13 | M/78 | Solid Neoplasia | No | Lung | pos | pos | pos/ *A.fumigatus* | neg | IA | *A.fumigatus* | n/a |
| PT14 | M/55 | ALL | Yes | Lung | pos | pos | neg | neg | n/a | n/a | *Aspergillus spp* |
| PT15 | F/52 | NHL,  alloSCT | Yes | Lung | pos | pos | pos/ *A.fumigatus* | pos | n/a | n/a | *Aspergillus spp* |
| PT16 | F/18 | ALL | Yes | Lung | n/a | pos | neg | neg | IA | *Aspergillus spp.* | n/a |
| PT17 | F/42 | AML,  alloSCT | Yes | Lung | pos | pos | neg | neg | IA | *A.fumigatus* | n/a |
| PT18 | M/55 | AML | Yes | Lung | pos | pos | neg | neg | IA | *Aspergillus spp* | n/a |
| PT19 | M/17 | ALL | No | Lung | n/a | pos | neg | neg | IA | *Aspergillus spp* | n/a |
| PT20 | F/25 | AML | Yes | Lung, sinus | pos | pos | pos/ *A.fumigatus* | pos | IA | *A.fumigatus* | n/a |

Abbreviations: AML= Acute Myeloid Leukemia; LLA= Acute Lymphoblastic Leukemia; CLL= Chronic Lymphoblastic Leukemia; NHL= non-Hodgkin’s lymphoma; SOT= Solid Organ Transplant; alloSCT= allogeneic stem cell transplant; neg= negative; pos= positive; n/a= not available; IA= invasive aspergillosis
