# Supplementary material for: Identification and validation of diagnostic cut-offs of the ELISpot assay for the diagnosis of invasive aspergillosis in high-risk patients
Source: PLoS One. 2024 Jul 9;19(7):e0306728. doi: 10.1371/journal.pone.0306728 (PMC11233002; doi:10.1371/journal.pone.0306728)
Supplement: S2 Table — Abbrevations: Pos. = positive; uninf. = uninformative; HRCT = High-Resolution Computed-scan Tomography; AML = acute myeloid leukemia; MDS = myelodisplastic syndrome; HL = Hodgkin Lymphoma; NHL = non-Hodgkin lymphoma; ALL = Acute lymphoblastic leukemia; MM = multiple myeloma; ASCT = autologous stem cell tranplantation; alloSCT = allogeneic hematopoietic stem cell transplant; S. aureus = Staphylococcus aureus; NA = not applicable. (DOCX) [file pone.0306728.s002.docx]

|  | | | | | | | | | | | | **ELISpot Samples** | | | | |  |
| --- | --- | --- | --- | --- | --- | --- | --- | --- | --- | --- | --- | --- | --- | --- | --- | --- | --- |
| **n°** | **Sex** | **Age** | **Underlying disorder** | **CHT** | **Definite diagnosis** | **Antifungal prophylaxis** | ***Day of CT findings (from clinical symptoms)*** | **HRCT appearence** | **Radiological criteria for possible IA** | ***GMs*** | ***GM Bal*** | **Nr** | ***n° pos. (days from HRCT)*** | ***n° neg. (days)*** | **n° uninf.** |  | |
| 1 | F | 25 | AML | Induction | Proven IA | Posaconazole | +5 | Nodular lesions | Possible | 5,27 | 10 | 5 | 5 (+9), (+19), (+29), (+35), (+42) | 0 | 0 |  | |
| 2 | M | 50 | AML | Salvage | Probable IA | NA | +2 | Nodular lesions | Possible | 0,21 | 1,11 | 4 | 3 (+5), (+10), (+16) | 1 (-1) | 0 |  | |
| 3 | M | 72 | AML | Salvage | Probable IA | NA | +3 | Nodular lesion surrounded by ground-glass | Possible | 0,09 | 1,04 | 4 | 4 (+4), (+11), (+18), (33) | 0 | 0 |  | |
| 4 | F | 45 | AML | Salvage | Probable IA | Posaconazole | +7 | Nodular lesion | Possible | 0,09 | 1,22 | 3 | 2 | 0 | 1 |  | |
| 5 | M | 40 | ALL | Induction | Probable IA | no | +5 | Nodular lesion surrounded by ground-glass opacities | Possible | 2,4 | 1,8 | 4 | 2 (+17), (+39) | 2 (+11), (+25) | 0 |  | |
| 6 | M | 64 | AML | Induction | Probable IA | Posaconazole | +1 | Nodular lesion surrounded by ground-glass opacities | Possible | 0,12 | 1,48 | 3 | 0 | 2 (+4), (+11) | 1 (-6) |  | |
| 7 | F | 23 | ALL | Induction | Bacterial pneumonia | no | +4 | Patchy consolidation with air bronchogram | no | 0,11 | 0,75 | 5 | 0 | 4 (+2), (+8), (+13), (+21) | 1(-6) |  | |
| 8 | F | 39 | AML | Consolidation | Possible IFD | Posaconazole | +4 | Nodular lesion surrounded by ground-glass opacities | Possible | 0,09 | 0,26 | 5 | 5 (+1), (+6), (+13), (+20), (+28) | 0 | 0 |  | |
| 9 | M | 72 | AML | Induction | Possible IFD | Posaconazole | +4 | Nodular lesion surrounded by ground-glass opacities | Possible | 0,08 | 0,1 | 4 | 2(+3), (+10) | 2 (+17), (+25) | 0 |  | |
| 10 | F | 58 | AML | Consolidation | Possible IFD | Posaconazole | +4 | Nodular lesion surrounded by ground-glass opacities | Possible | 0,09 | 0,05 | 3 | 0 | 3 (+16), (+31), (+43) | 0 |  | |
| 11 | F | 69 | AML | Salvage | Parainfluenza 1, S.mitis (BSI) | Posaconazole | +2 | Tree-in-bud pattern | Possible | 0,13 | 0,19 | 3 | 0 | 3 (+8), (+23), (+36) | 0 |  | |
| 12 | M | 64 | AML | Induction | Possible IFD | Posaconazole | +1 | Nodular lesion surrounded by ground-glass opacities | Possible | 0,11 | 0,22 | 4 | 1(+13) | 3 (+7), (+20), (+28) | 0 |  | |
| 13 | F | 22 | AML | Salvage | NA | Posaconazole | NA | NA | No | 0,1 | 0,1 | 3 | 2 | 0 | 1 |  | |
| 14 | M | 55 | AML | Salvage | NA | Posaconazole | NA | NA | No | 0,1 | 0,1 | 3 | 0 | 2 | 1 |  | |
| 15 | F | 51 | AML | Consolidation | No | Posaconazole | +16 | ground glass opacity | No | 0,11 | 0,1 | 2 | 0 | 2 (+44), (+74) | 0 |  | |
| 16 | M | 68 | NHL | Induction | S.maltophilia | No | +7 | ground glass opacity | No | 0,14 | 0,53 | 3 | 0 | 3 (+6), (+13), (+20) | 0 |  | |
| 17 | M | 21 | ALL | Consolidation | C.difficile | NO | No scan performed | No scan performed | No | 0,09 | Not performed | 3 | 0 | 3 | 0 |  | |
| 18 | M | 26 | AML | Consolidation | No | Posaconazole | No scan performed | No scan performed | No | 0,09 | Not performed | 2 | 0 | 2 | 0 |  | |
| 19 | M | 26 | HL | alloSCT | viral infection | Posaconazole | No scan performed | No scan performed | No | 0,11 | Not performed | 1 | 0 | 1 | 0 |  | |
| 20 | M | 71 | AML | Induction | Bacterial pneumonia | Posaconazole | +4 | micronodular | No | 0,34 | Not performed | 3 | 0 | 3 (+8), (+12), (+38) | 0 |  | |
| 21 | F | 63 | AML | Induction | Bacterial pneumonia | Posaconazole | +11 | micronodular | No | 0,07 | Not performed | 3 | 1 (-9) | 2 (+9), (+24) | 0 |  | |
| 22 | F | 64 | AML | Induction | Bacterial pneumonia | Posaconazole | +2 | CT negative | No | 0,07 | 0,09 | 3 | 0 | 2(+6), (18) | 1(-1) |  | |
| 23 | F | 64 | AML | Induction | NA | Posaconazole | NA | NA | No | 0,12 | Not performed | 2 | 2 | 0 | 0 |  | |
| 24 | M | 27 | ALL | Induction | No | no | +4 | Interstitial | No | 0,12 | 0,18 | 3 | 1 (+26) | 2 (+4), (+33) | 0 |  | |
| 25 | M | 50 | AML | Induction | Obliterative bronchiolitis | Posaconazole | +6 | Interstitial | No | 0,17 | 0,18 | 3 | 1 (+27) | 1 (+114) | 1 (+14) |  | |
| 26 | F | 51 | AML | alloSCT | NA | Posaconazole | NA | NA | No | 0,1 | NA | 2 | 0 | 2 | 0 |  | |
| 27 | M | 55 | AML | Consolidation | S.aureus | Posaconazole | +3 | Nodular lesion surrounded by ground-glass opacities | Possible | 0,27 | 0,3 | 2 | 0 | 2 (+12), (+17) | 0 |  | |
| 28 | F | 45 | AML | Induction | No | Posaconazole | +3 | CT negative | No | 0,07 | Not performed | 1 | 0 | 1 (-2) | 0 |  | |
| 29 | M | 49 | ALL | Consolidation | E.coli (BSI) | no | No scan performed | No scan performed | No | 0,14 | Not performed | 2 | 0 | 2 | 0 |  | |
| 30 | M | 71 | AML | Consolidation | Streptococcus (BSI) | no | +4 | CT negative | no | 0,31 | Not performed | 2 | 0 | 2 (+5), (+19) | 0 |  | |
| 31 | M | 31 | AML | alloSCT | No | posaconazole | +8 | CT negative | No | 0,06 | Not performed | 3 | 0 | 3 (+18), (+22), (+112) | 0 |  | |
| 32 | F | 65 | AML | Salvage | No | posaconazole | No scan performed | No scan performed | No | 0,1 | Not performed | 3 | 0 | 1 | 2 |  | |
| 33 | M | 39 | ALL | Consolidation | Urinary tract infection | no | NA | micronodular | No | 0,07 | Not performed | 2 | 0 | 2 | 0 |  | |
| 34 | F | 61 | AML | Induction | No | Posaconazole | +6 | CT negative | No | 0,07 | Not performed | 2 | 0 | 2 (+4), (+19) | 0 |  | |
| 35 | M | 41 | ALL | Induction | No | Fluconazole | No scan performed | No scan performed | No | 0,07 | Not performed | 2 | 0 | 2 | 0 |  | |
| 36 | M | 27 | ALL | Induction | No | Fluconazole | No scan performed | No scan performed | No | 0,1 | Not performed | 3 | 0 | 3 | 0 |  | |
| 37 | M | 69 | AML | Consolidation | S.aureus | Fluconazole | +7 | CT negative | no | 0,13 | 0,11 | 2 | 0 | 2 (+57), (+71) | 0 |  | |
| 38 | M | 55 | AML | Salvage | S.aureus | posaconazole | NA | Multiple nodular lesions | Possible | 0,2 | 0,3 | 3 | 0 | 3 | 0 |  | |
| 39 | F | 73 | AML | Induction | Viral pneumonia | posaconazole | No scan performed | No scan performed | No | 0,09 | Not performed | 2 | 0 | 2 | 0 |  | |
| 40 | M | 75 | AML | Salvage | S.epidermidis (BSI) | posaconazole | +1 | micronodular | No | 0,15 | 0,78 | 2 | 0 | 2 | 0 |  | |
| 41 | M | 39 | ALL | Induction | E.coli (BSI) | posaconazole | +1 | CT negative | No | 0,1 | Not performed | 3 | 0 | 3(+1), (+12), (+26) | 0 |  | |
| 42 | F | 56 | AML | Induction | Salivary Gland Bacterial infection | posaconazole | No scan performed | No scan performed | No | 0,09 | Not performed | 3 | 0 | 3 | 0 |  | |
| 43 | M | 52 | AML | Induction | Viral pneumoniae | posaconazole | +7 | Interstitial | No | 0,09 | 0,34 | 3 | 0 | 3 (-2), (+12), (+25) | 0 |  | |
| 44 | M | 49 | AML | alloSCT | Atipical mycobacterial disease | posaconazole | +14 | Nodular lesions | Possible | 0,13 | 0,69 | 4 | 0 | 4(-10), (+1), (+15), (+30) | 0 |  | |
| 45 | M | 27 | ALL | Consolidation | Typhlitis | posaconazole | No scan performed | No scan performed | No | 0,17 | Not performed | 3 | 0 | 3 | 0 |  | |
| 46 | M | 19 | ALL | Induction | Viral pneumoniae | no | +2 | Interstitial | No | 0,08 | 0,15 | 3 | 0 | 2 | 1 |  | |
| 47 | F | 62 | AML | Induction | BSI | posaconazole | No scan performed | No scan performed | No | 0,1 | Not performed | 3 | 0 | 2 | 1 |  | |
| 48 | M | 48 | AML | Induction | S.aureus | posaconazole | +3 | Patchy consolidation | no | 0,23 | 0,48 | 3 | 0 | 2(+24), (+39) | 1(+14) |  | |
| 49 | F | 74 | AML | Induction | No | posaconazole | No scan performed | No scan performed | No | 0,07 | Not performed | 3 | 0 | 2 | 1 |  | |
| 50 | M | 73 | AML | Induction | No | posaconazole | No scan performed | No scan performed | No | 0,1 | Not performed | 3 | 0 | 2 | 1 |  | |
| 51 | M | 41 | ALL | Consolidation | No | posaconazole | No scan performed | No scan performed | no | 0,12 | Not performed | 3 | 0 | 3 | 0 |  | |
| 52 | M | 65 | AML | Induction | No | posaconazole | +1 | CT negative | No | 0,1 | Not performed | 3 | 0 | 2 (+20), (+34) | 1(+7) |  | |
| 53 | M | 51 | AML | alloSCT | No | posaconazole | +2 | Interstitial | No | 0,08 | 0,08 | 2 | 0 | 1 (+43) | 1(+2) |  | |
| 54 | M | 64 | AML | Induction | No | posaconazole | +3 | interstitial | No | 0,07 | Not performed | 3 | 0 | 3 (+4), (+20), (+42) | 0 |  | |
| 55 | M | 60 | AML | Induction | Viral pneumoniae | posaconazole | +4 | Interstitial | No | 0,22 | 0,17 | 3 | 0 | 3 (+1), (+11), (+19) | 0 |  | |
| 56 | M | 21 | ALL | Induction | No | Fluconazole | +1 | micronodular | No | 0,07 | Not performed | 4 | 0 | 4 (-8), (+6), (+20), (+28) | 0 |  | |
| 57 | F | 38 | ALL | Induction | No | Fluconazole | NA | No scan performed | no | 0,07 | Not performed | 3 | 0 | 3 | 0 |  | |
| 58 | F | 65 | AML | Induction | Viral pneumoniae | posaconazole | +2 | Nodular lesion | Possible | 0,08 | 0,19 | 3 | 0 | 1(+15) | 2(+3), (+22) |  | |
| 59 | F | 70 | AML | Salvage | No | posaconazole | No scan performed | No scan performed | No | 0,09 | Not performed | 3 | 0 | 3 | 0 |  | |
| 60 | M | 52 | ALL | Salvage | No | Posaconazole | No scan performed | No scan performed | No | 0,1 | Not performed | 3 | 0 | 3 | 0 |  | |
| 61 | F | 55 | ALL | Consolidation | No | Fluconazole | No scan performed | No scan performed | No | 0,07 | Not performed | 3 | 0 | 2 | 1 |  | |
| 62 | M | 20 | Aplastic anemia | alloSCT | No | posaconazole | No scan performed | No scan performed | No | 0,08 | Not performed | 2 | 0 | 1 | 1 |  | |
| 63 | M | 48 | ALL | Induction | Viral pneumoniae | Fluconazole | No scan performed | No scan performed | No | 0,07 | Not performed | 3 | 0 | 3 | 0 |  | |
| 64 | F | 31 | AML | Consolidation | E.coli (BSI) | Posaconazole | No scan performed | No scan performed | No | 0,1 | Not performed | 2 | 0 | 2 | 0 |  | |
| 65 | M | 73 | AML | Induction | P.aeruginosa | posaconazole | +1 | Patchy consolidation | No | 0,17 | 0,26 | 3 | 0 | 2 (+16), (+37) | 1 (-4) |  | |
| 66 | M | 69 | AML | Salvage | Fusarium | posaconazole | +3 | CT negative | No | 0,07 | Not performed | 3 | 1 (-2) | 2 (+4), (+12) | 0 |  | |
| 67 | M | 65 | AML | Salvage | No | posaconazole | +2 | CT negative | No | *0,08* | Not performed | 2 | 0 | 1 (+27) | 1 (+13) |  | |
| 68 | M | 48 | ALL | Induction | C.glabrata | Fluconazole | NA | NA | No | *0,1* | NA | 1 | 0 | 1 | 0 |  | |
| 69 | M | 44 | MM | ASCT | P.aeruginosa (BSI) | posaconazole | No scan performed | No scan performed | No | *0,12* | Not performed | 2 | 0 | 2 | 0 |  | |
| 70 | F | 47 | AML | Induction | No | posaconazole | No scan performed | No scan performed | No | *0,11* | Not performed | 3 | 0 | 2 | 1 |  | |
| 71 | M | 73 | AML | Induction | BSI | posaconazole | No scan performed | No scan performed | No | *0,07* | Not performed | 3 | 0 | 2 | 1 |  | |
| 72 | M | 73 | AML | Induction | No | posaconazole | No scan performed | No scan performed | No | *0,1* | Not performed | 2 | 0 | 2 | 0 |  | |
| 73 | M | 43 | AML | Induction | No | posaconazole | +2 | CT negative | No | *0,09* | Not performed | 2 | 0 | 2 (+5), (+48) | 0 |  | |
| 74 | M | 47 | AML | Salvage | BSI | posaconazole | +6 | CT negative | no | *0,12* | Not performed | 2 | 1 (+4) | 1 (+17) | 0 |  | |
| 75 | F | 19 | Aplastic Anemia | Induction | No | posaconazole | No scan performed | No scan performed | No | *0,1* | Not performed | 2 | 0 | 2 | 0 |  | |
| 76 | F | 24 | AML | Induction | No | posaconazole | No scan performed | No scan performed | No | *0,11* | Not performed | 3 | 1 | 1 | 1 |  | |
| 77 | F | 69 | AML | Induction | No | posaconazole | +4 | CT negative | No | *0,1* | Not performed | 3 | 1 (+20) | 1(+11) | 1 (-3) |  | |
| 78 | M | 40 | CML | alloSCT | No | posaconazole | No scan performed | No scan performed | No | *0,13* | Not performed | 3 | 2 | 1 | 0 |  | |
| 79 | M | 80 | ALL | Induction | No | no | No scan performed | No scan performed | No | *0,13* | Not performed | 3 | 3 | 0 | 0 |  | |
| 80 | M | 69 | AML | Induction | No | Posaconazole | No scan performed | No scan performed | No | *0,16* | Not performed | 3 | 2 | 0 | 1 |  | |
| 81 | M | 32 | HSCT | HSCT | No | voriconazole | NA | NA | No | *0,08* | NA | 2 |  | 1 | 1 |  | |
| 82 | F | 65 | AML | induction | C.glabrata | fluconazole | NA | NA | no | *0,3* | NA | 3 | 0 | 3 | 0 |  | |
| 83 | F | 61 | AML | Salvage | No | posaconazole | NA | NA | no | *0,07* | NA | 2 | 0 | 2 | 0 |  | |
| 84 | M | 50 | MDS | alloSCT | Viral pneumoniae | posaconazole | NA | NA | no | *0,07* | NA | 2 | 0 | 1 | 1 |  | |
| 85 | F | 50 | AML | Salvage | BSI | posaconazole | NA | NA | no | *0,1* | NA | 3 | 0 | 1 | 2 |  | |
| 86 | M | 50 | HL | alloSCT | No | posaconazole | NA | NA | No | *0,11* | NA | 3 | 0 | 1 | 2 |  | |
| 87 | M | 52 | AML | alloSCT | No | voriconazole | NA | NA | No | *0,12* | NA | 3 | 0 | 3 | 0 |  | |
| 88 | M | 39 | AML | Salvage | Viral pneumoniae | posaconazole | NA | NA | no | *0,23* | NA | 3 | 0 | 2 | 1 |  | |
| 89 | F | 22 | AML | Salvage | No | posaconazole | NA | NA | No | *0,05* | NA | 3 | 0 | 2 | 1 |  | |
| 90 | F | 60 | AML | alloSCT | No | posaconazole | NA | NA | No | *0,07* | NA | 3 | 0 | 2 | 1 |  | |
| 91 | F | 63 | AML | alloSCT | No | posaconazole | NA | NA | No | *0,14* | NA | 3 | 0 | 2 | 1 |  | |
| 92 | F | 73 | AML | Salvage | No | posaconazole | NA | NA | No | *0,07* | NA | 3 | 0 | 2 | 1 |  | |
| 93 | M | 45 | AML | Induction | Viral | posaconazole | NA | NA | No | *0,07* | NA | 1 | 0 | 1 | 0 |  | |
| 94 | F | 53 | AML | Induction | No | posaconazole | NA | NA | No | *0,13* | NA | 3 | 0 | 2 | 1 |  | |
| 95 | F | 52 | AML | Induction | No | posaconazole | NA | NA | No | *0,1* | NA | 3 | 0 | 2 | 1 |  | |
| 96 | M | 65 | AML | Consolidation | No | No | NA | NA | No | *0,1* | NA | 1 | 0 | 1 | 0 |  | |
| 97 | F | 63 | AML | Salvage | Viral pneumoniae | Posaconazole | NA | NA | No | *0,11* | NA | 2 | 0 | 1 | 1 |  | |
| 98 | M | 59 | AML | alloSCT | No | Posaconazole | NA | NA | No | *0,2* | NA | 2 | 0 | 1 | 1 |  | |
| 99 | M | 58 | AML | Induction | Fusarium | Posaconazole | NA | CT negative | No | *0,07* | Not performed | 2 | 0 | 2 | 0 |  | |
| 100 | M | 46 | AML | Induction | Fusarium | Posaconazole | NA | CT negative | No | *0,08* | Not performed | 2 | 0 | 2 | 0 |  | |

Abbreviations: Pos. = positive; uninf. = uninformative; HRCT = High-Resolution Computed-scan Tomography; AML = acute myeloid leukemia; MDS = myelodisplastic syndrome; HL= Hodgkin Lymphoma; NHL = non-Hodgkin lymphoma; ALL = Acute lymphoblastic leukemia; MM= multiple myeloma; ASCT= autologous stem cell tranplantation; alloSCT = allogeneic hematopoietic stem cell transplantation; S. aureus = Staphylococcus aureus; BSI= Blood stream Infection; n.a. = not applicable.
