# Supplementary figures and images for: Identification and validation of diagnostic cut-offs of the ELISpot assay for the diagnosis of invasive aspergillosis in high-risk patients
Source: PLoS One. 2024 Jul 9;19(7):e0306728. doi: 10.1371/journal.pone.0306728 (PMC11233002; doi:10.1371/journal.pone.0306728)

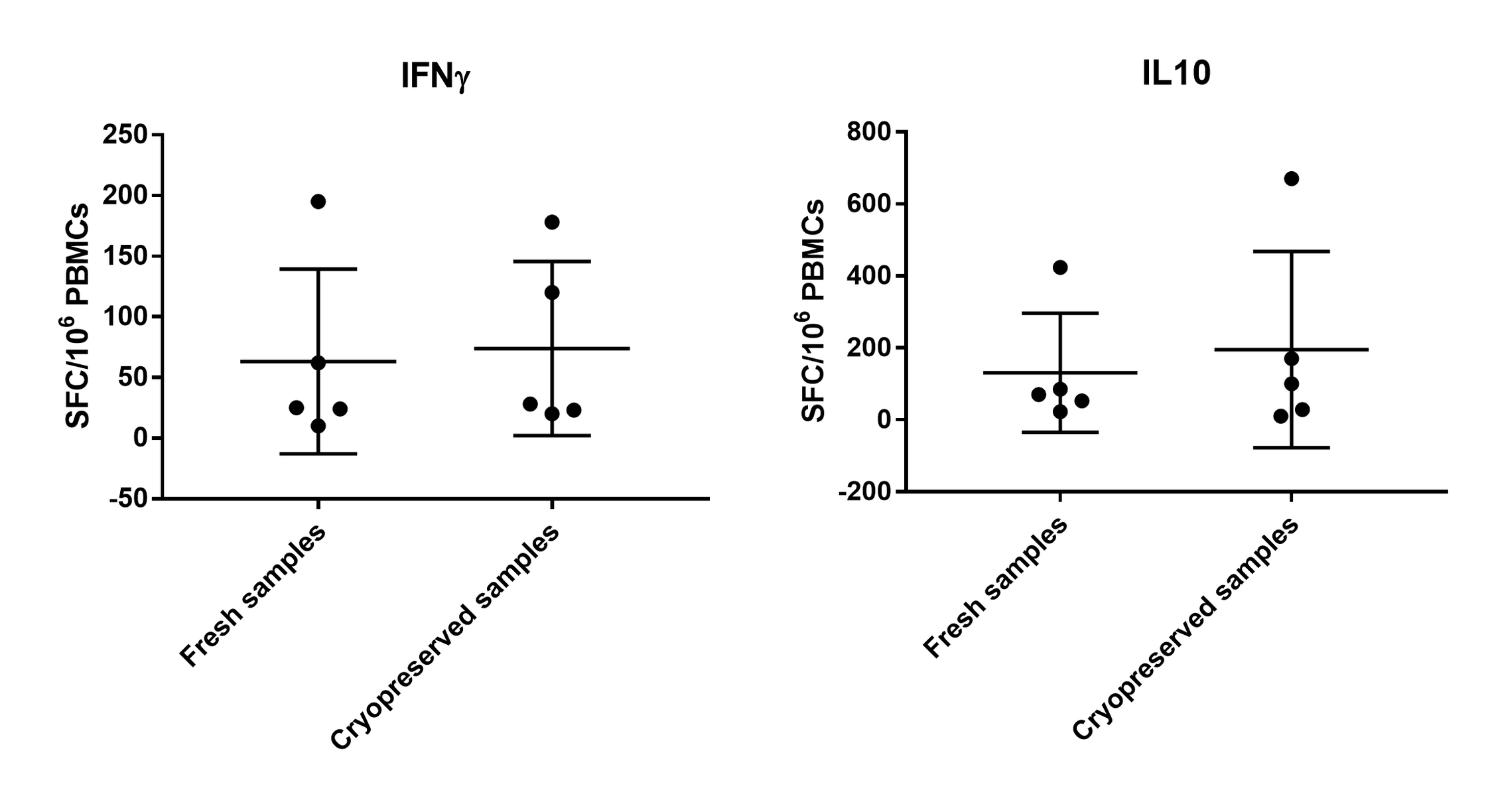

Supplement: S1 Fig — Results are expressed as the number of SFC per 106 PBMCs. Mean with SD is given. (TIF) [file pone.0306728.s003.tif]
