# Supplementary material for: Identification and validation of diagnostic cut-offs of the ELISpot assay for the diagnosis of invasive aspergillosis in high-risk patients
Source: PLoS One. 2024 Jul 9;19(7):e0306728. doi: 10.1371/journal.pone.0306728 (PMC11233002; doi:10.1371/journal.pone.0306728)
Supplement: S1 File — (DOCX) [file pone.0306728.s004.docx]

**PBMC cryopreservation**

Upon counting, cells were suspended in 50% FBS, 30% RPMI 1640 and 20% DMSO at a concentration of 20-30x10^6^ cells per 900 μl. In detail, FBS was added immediately and a mix of RPMI and DMSO was added drop by drop in ice. The 900 μl aliquots were transferred in cryovials and frozen at −80°C in a freezing container (Nalge Nunc International, Rochester, NY, USA). After 24-72 hours, cells were transferred to liquid nitrogen for long-term storage.
