# Supplementary material for: Identification and validation of diagnostic cut-offs of the ELISpot assay for the diagnosis of invasive aspergillosis in high-risk patients
Source: PLoS One. 2024 Jul 9;19(7):e0306728. doi: 10.1371/journal.pone.0306728 (PMC11233002; doi:10.1371/journal.pone.0306728)
Supplement: S2 File — (DOCX) [file pone.0306728.s005.docx]

**PBMC thawing**

Cryopreserved PBMCs were thawed in a water bath at 37°C. DNase I (Roche diagnostics, GmbH, Germany) was added at 1 mg/ml concentration to avoid cell clumping. Cell suspension was transferred to a 15 ml centrifugation tube and pre-warmed thawing medium (R10) was added dropwise up to a total volume of 12 ml, gently shaking sometimes. After centrifugation for 10 min at 250 g 1200 rpm, supernatants were discarded and cells were washed in 12 ml pre-warmed RPMI medium, followed by another centrifugation step (10 min, 250 g 1200 rpm). Cell viability after thawing was evaluated by Trypan blue (Gibco) cell counting.

Upon counting, cells were diluted in R10 medium at the appropriate cell concentration (2-2.5x10^6^/ml) and rested over-night in 6-well plates at 37° C in a humidified 5% CO2 atmosphere. After resting, cells were transferred to a 15 ml centrifugation tube, washed in warmed RPMI medium, centrifuged for 10 min at 250 g 1200 rpm and counted. Cell viability after resting was also evaluated by Trypan blue (Gibco) cell counting.
